# Nasopharyngeal Cancer Outcomes From Saudi Arabia

**DOI:** 10.1200/JGO.2016.003418

**Published:** 2016-05-18

**Authors:** Manikandan Dhanushkodi

**Affiliations:** Cancer Institute (W.I.A.), Chennai, India

## To the Editor:

In their recent article in *Journal of Global Oncology*, Al-Wassia et al^1^ reported on the outcomes of Saudi Arabian patients with nasopharyngeal cancer treated with primarily neoadjuvant chemotherapy followed by concurrent chemoradiotherapy. We have a few questions about the study.

Why was neoadjuvant chemotherapy given when the meta-analysis of chemotherapy in nasopharyngeal cancer ^2^ clearly showed no benefit? What were the doses of the docetaxel, cisplatin, 5-fluorouracil regimen used as neoadjuvant therapy? How were the patients selected for cisplatin either 100 mg/m^2^ every 3 weeks or 30 mg/m^2^ once per week? Did patients receive adjuvant chemotherapy?

On what basis were patients selected for neoadjuvant chemotherapy followed by concurrent chemoradiotherapy, neoadjuvant chemotherapy followed by radiotherapy, or concurrent chemoradiotherapy alone? Did any patients undergo resection of residual neck nodes after completion of therapy? How were the patients with relapse managed?

Finally, was Epstein-Barr virus testing performed and was there any association with tobacco or alcohol consumption?
